# Characterising viable virus from air exhaled by H1N1 influenza-infected ferrets reveals the importance of haemagglutinin stability for airborne infectivity

**DOI:** 10.1371/journal.ppat.1008362

**Published:** 2020-02-25

**Authors:** Anika Singanayagam, Jie Zhou, Ruth A. Elderfield, Rebecca Frise, Jonathan Ashcroft, Monica Galiano, Shahjahan Miah, Laura Nicolaou, Wendy S. Barclay

**Affiliations:** 1 Department of Medicine, St. Marys Campus, Imperial College, London, United Kingdom; 2 Virus Reference Department, Public Health England, Colindale, United Kingdom; 3 Department of Mechanical Engineering, Johns Hopkins University, Baltimore, Maryland, United States of America; Emory University School of Medicine, UNITED STATES

## Abstract

The transmissibility and pandemic potential of influenza viruses depends on their ability to efficiently replicate and be released from an infected host, retain viability as they pass through the environment, and then initiate infection in the next host. There is a significant gap in knowledge about viral properties that enable survival of influenza viruses between hosts, due to a lack of experimental methods to reliably isolate viable virus from the air. Using a novel technique, we isolate and characterise infectious virus from droplets emitted by 2009 pandemic H1N1-infected ferrets. We demonstrate that infectious virus is predominantly released early after infection. A virus containing a mutation destabilising the haemagglutinin (HA) surface protein displayed reduced survival in air. Infectious virus recovered from droplets exhaled by ferrets inoculated with this virus contained mutations that conferred restabilisation of HA, indicating the importance of influenza HA stability for between-host survival. Using this unique approach can improve knowledge about the determinants and mechanisms of influenza transmissibility and ultimately could be applied to studies of airborne virus exhaled from infected people.

## Author summary

Influenza viruses can transmit through the air between two hosts. For virus to successfully transmit through the air, it must be exhaled from an infected donor in sufficient quantities and retain infectiousness in the air. These aspects of transmission are poorly understood due to a paucity of methods for quantifying infectious virus from airborne particles. Using a novel technique of virus plaque isolation from depositing airborne droplets, we show that ferrets infected with an airborne transmissible influenza virus exhaled a peak of infectious virus early after infection. We demonstrate the importance of virion stability for the retention of infectivity as virus travels through the air. Our findings highlight the fate of infectious virus outside the respiratory tract as an important parameter for understanding influenza transmission.

## Introduction

Risk assessing an influenza virus for sustained human-to-human transmission is key to understanding its pandemic potential and the threat to public health[1]. Animal models, particularly ferrets, are extensively used to evaluate the capacity of influenza viruses to transmit through the air. Typically, naïve sentinel ferrets are housed in cages adjacent to an infected donor allowing only for transmission of virus released into the air. The focus of these experiments is on the ability of virus to replicate in the respiratory tract of the donor animal and its ability to initiate infection in a recipient. However, for virus to successfully transmit through the air between two hosts, it must be exhaled from the donor in sufficient quantities and retain infectiousness in the air. A critical unknown in the field is how much infectious virus a ferret (or human) releases into the air and how viability is affected as virus travels between hosts, within droplets and aerosols.

Infectious virus is released into the air from an infected donor into particles of different sizes[2–5]. Practically, The Infectious Diseases Society of America define “respirable” particles as those <10μm that can deposit in both lower and upper airways and “inspirable” particles as those 10–100μm that predominantly deposit in upper airways[6]. Particles released into the air may also fall to ground and remain infectious to an onward host as contaminated fomites for a period of time. Droplet behaviour in the air can be inferred by size to some extent but is also affected by factors such as airflow streams, humidity and temperature[7]. Hereafter, we use the term “aerosol” to indicate particles <10μm including droplet nuclei resulting from dessication of larger droplets, that may travel both short and longer distances. We use the term “droplets” to indicate both large particles (>20μm) that are likely to follow a more ballistic trajectory as well as intermediate particles (10–20μm) that are described to share properties of both small and large particles[7].

Virus infectivity in the air is likely to be dependent on the constituents of respiratory secretions at the time of emission and molecular properties of the infecting viral strain. One viral property demonstrated to facilitate transmission in the ferret model is stability of the haemagglutinin (HA) surface protein[8–10]. Some avian influenza viruses, such as H5N1 and H7N9, have less stable HA proteins and this appears to be an important factor restricting their zoonotic potential[11]. We hypothesised that increased HA stability would facilitate transmission by enhancing virus survival in airborne droplets/aerosols. To investigate, we developed a novel technique for isolating and characterising infectious virus that is released into the air, involving the collection of depositing virus plaques on cell culture plates. Here, we describe the use of this technique to demonstrate that infected ferrets emit the majority of contagious virus into the air early after infection, with decreasing amounts of infectious virus detected over the course of infection. We find that HA stability is critical for retention of infectivity in airborne droplets, providing a mechanistic understanding for previous reports of the requirement of this viral property for transmission[8–10].

## Results

### Establishing a system to collect infectious influenza virus from airborne droplets

**Fig 1:**
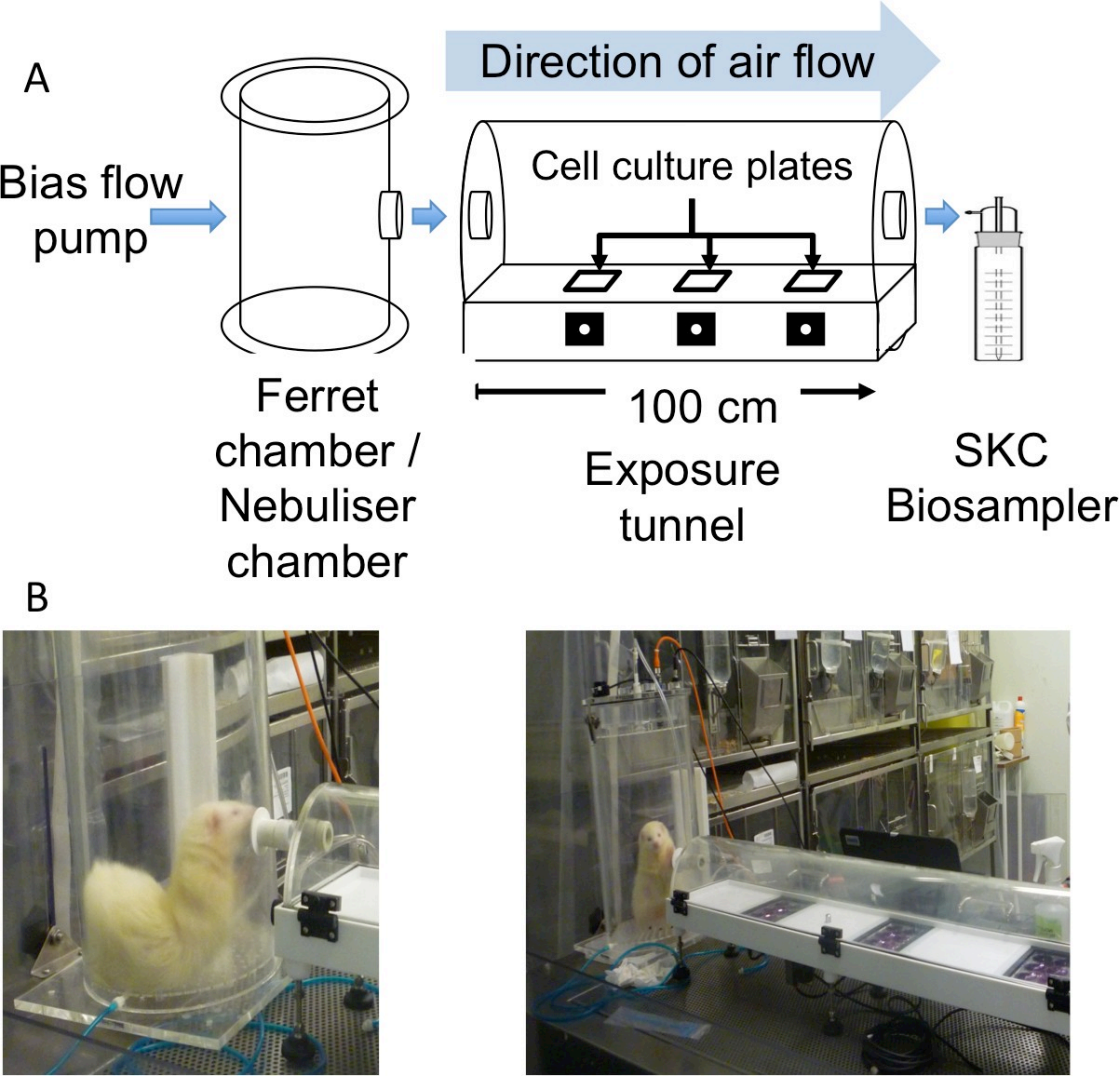
The influenza virus transmission tunnel (IVTT). (A) Schematic diagram of the IVTT apparatus. Airflow is generated using a bias flow pump, which connects to a 37.5cm (height) x 25cm (diameter) ferret chamber for *in vivo* experiments, or a 10cm (height) x 9cm (diameter) nebuliser chamber for *in vitro* experiments with nebulised virus. The IVTT is a half cylindrical clear acrylic 100cm (length) x 18cm (width) x 9cm (height) exposure tunnel containing cell culture plates situated 30cm, 60cm and 90cm from the tunnel opening. Air can be sampled from the end of the IVTT into a SKC Biosampler. (B) Photographs of the IVTT in use for isolating virus from an influenza virus-infected ferret.

Previous studies have attempted quantification of influenza virus from respiratory droplets and/or aerosols using bioaerosol samplers or impactors and assaying for viral nucleic acid[2–4,12–15]. However, due to limitations inherent to these sampling approaches, such as shear forces that can damage virions[16], the infectiousness of air samples cannot be reliably demonstrated. We designed a bespoke device capable of sampling infectious influenza virus released into the air. The influenza virus transmission tunnel (IVTT) consists of a 100cm (length) x 18cm (width) x 9cm (height) half-cylindrical clear acrylic exposure tunnel that can hold three plates of susceptible cells at different distances from the source. Virus is introduced into one end of the tunnel either using a nebulisation unit or via breath from an infected animal and directional airflow maintained by a bias flow pump (Fig 1A and 1B). Virus-laden droplets falling onto open culture plates during a 10-minute collection period are detected as viral plaques, enabling quantification and further analysis. The use of direct viral plaque collection is a far more sensitive method of detection for viable virus than existing impaction techniques and has the key advantage of enabling isolation and characterisation of individual depositing viruses. Further details of the experimental set up are described in the Methods section.

Within the IVTT, droplets collected are likely to be of a certain minimum size in order to settle the short distance (~5cm) onto cell culture plates during the 10-minute sampling period. To obtain an estimate of particle sizes depositing, we adopted a model for the airflow, motion and evaporation of particles in the IVTT (see Methods section for details). With this model, we make a conservative estimate that particles with diameters ≥7.8μm are collected. To understand how airborne particles distribute within the tunnel we nebulised a 2009 human pandemic H1N1 (pH1N1) virus mixed with a DNA plasmid tracer into the IVTT. The majority of infectious viral plaques were collected on the first exposed plate of cells (situated 30cm from the tunnel opening), with a decline in plaque count seen by plate 2 (at 60cm) and plate 3 (at 90cm) Following 10-minutes exposure of cell culture plates to nebulised particles, we sampled any non-sedimenting aerosols from the air within the IVTT into an SKC Biosampler. The SKC Biosampler, which can collect particles with aerodynamic diameter of 0.3μm to 8μm[17,18], is a device that has been previously used for collection of viable airborne influenza viruses and cited to be one of the more efficient available methods[19], being used as a standard with which to compare other techniques[20–22]. No infectious virus was collected from the air sample, whereas tracer DNA and viral RNA were detected (S1 Fig). The reason that infectious virus was not collected is likely due to virion damage during the sampling process, although it is possible that influenza virus viability is not well maintained in small aerosols, as has been suggested in some previous reports[2,3,23]. Taken together our results demonstrate that the IVTT is capable of sampling viable virus from airborne droplets but, as found in previous studies, the efficiency of collection of viable virus from aerosols and droplet nuclei with gravitational settling times of >10 minutes using an air sampling device, is less certain.

### The IVTT system can detect infectious virus emitted by ferrets infected with an airborne transmissible influenza virus

**Fig 2:**
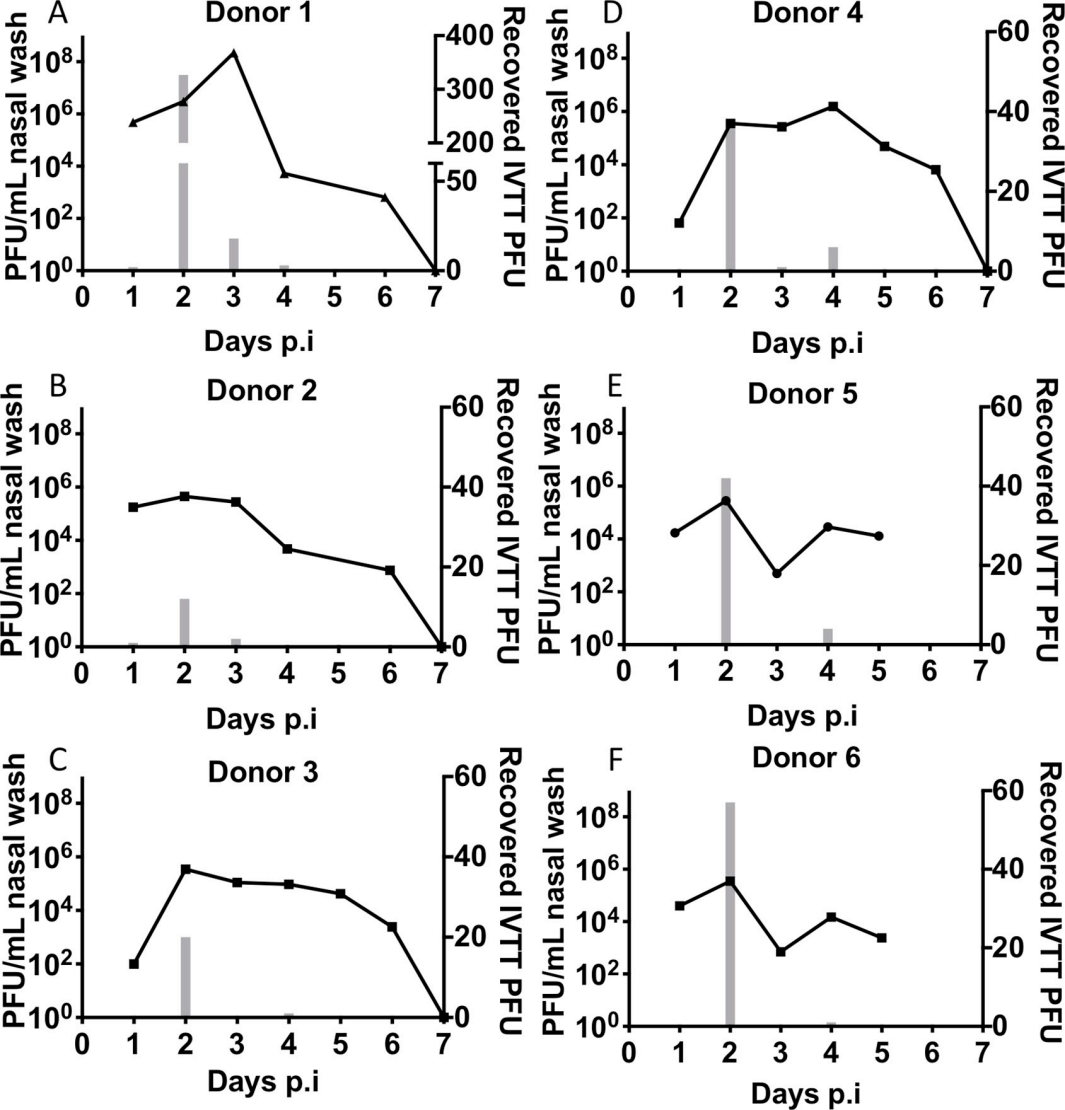
Ferrets emit a peak of infectious virus early after infection. Ferrets were intranasally inoculated with 10^4^ PFU of Eng/09 virus diluted in 0.2mL PBS. On days 1 to 6, air was sampled for 10 minutes using the IVTT and then each ferret was nasal washed while conscious. Virus titre (PFU/mL) detected by plaque assay from nasal wash samples is shown as lines in (A) to (F) on the left y-axis. Total numbers of viral plaques (PFU) detected in the IVTT is shown as grey bars in (A) to (F) on the right y-axis.

The limitations of *in vitro* experiments using nebulised virus are that airborne particles will not necessarily display the same size distribution nor have the same composition of salts/proteins/lipids as from respiratory secretions and thus virus may display a different lifespan to that occurring in an *in vivo* setting. We therefore focussed our efforts on detection of infectious virus emitted by infected ferrets. In three independent experiments, two ferrets were intranasally inoculated with pH1N1 virus (Eng/09). We[24–26] and others[27,28], have previously shown highly efficient transmission between ferrets infected with pH1N1. All six infected animals shed robust titres of virus in the nasal wash from day 1 post inoculation (p.i.) until day 5/6. The clinical illness induced was mild with limited weight loss, coughs, sneezes and mucous production. We successfully detected infectious virus in the IVTT on day 2 p.i. shed from all ferrets and to a lesser extent on day 3/4 (Fig 2). No ferrets were observed to cough or sneeze during the 10-minute breath collection on day 2 when the majority of virus was detected in the IVTT. As seen for nebulised virus, the number of plaques detected declined with increasing distance along the IVTT. Nonetheless, infectious virus emitted from 5 out of 6 ferrets was detected on plate 3, 90cm from the ferret chamber (S2 Fig). After adjustments for sampling efficiency (see conversion calculation in Methods section), we estimated the amount of infectious virus emitted by pH1N1-infected ferrets within 10 minutes to be at least 72 PFU and as much as 1388 PFU at the peak of detection on day 2 p.i.

There was a clear peak in airborne infectious virus on day 2 for all ferrets. This correlated with peak nasal wash titre in some ferrets but in others the timing was discordant. For donors #1 and #4, peak virus shedding in the nasal wash was on day 3 and 4 respectively when a low number of plaques were detected in the IVTT, but an order of magnitude less than was detected on day 2 (Fig 2). On other days where a substantial amount of virus was present in the nasal wash, there was no viable airborne virus detected. This data demonstrates that the typically used measurement of nasal virus does not reliably predict the infectivity of virus that is released into the air from infected animals and should not be used as a surrogate for contagiousness. Replication in the nasal cavity is likely a requirement for transmission but not sufficient for transmission to occur.

### Ferrets infected with a stable pH1N1 HA mutant release more infectious virus into the air

We next sought to implement our methodology to investigate how virus viability in airborne droplets might vary between influenza viruses with different pH stability. A pH stable HA surface protein was previously demonstrated to facilitate successful transmission of virus through the air between ferrets[8–10]. Influenza HA is involved in virus-host fusion during cell entry and this process involves a pH-dependent conformational change in HA that allows for exposure of the fusogenic machinery[29]. If HA is triggered to unfold before entering a host cell, the virus is rendered non-infectious. We hypothesised that a more fragile HA could become inactivated during exposure to the microenvironment of airborne droplets, reducing the likelihood of viable virus travelling the distance to a recipient host.

We generated recombinant mutants of pH1N1 with HA mutations that alter its pH stability. Mutation Y7H (Y17H in H3 numbering) was previously shown to destabilise Eng/09 HA, increasing the pH of fusion to 5.9, a level similar to highly pathogenic avian H5N1 viruses that do not transmit via the airborne route. Mutation E21K (E31K in H3 numbering) was previously shown to stabilise Eng/09 HA, decreasing pH of fusion to 5.3, typical of a transmissible human seasonal virus. Both viruses readily formed plaques on MDCK cells and grew with similar replication kinetics in multicycle replication assays in MDCK cell monolayers. Details on the generation and analysis of these viruses have been reported previously[30].

We inoculated 4 ferrets each with either Y7H or E21K pH1N1 mutants and sampled using the IVTT on days 1 to 4 p.i. Two ferrets (donors #1 and #2) in each group were infected with 10^4^ PFU and two ferrets (donors #3 and #4) were infected with 10^6^ PFU. Based on our previous work that identified replicative differences between Y7H and E21K in primary airway cells[30], we predicted that Y7H might replicate in the ferret nose at lower titres than E21K. In order to achieve comparable titres of virus shed into the air, we elected to inoculate Y7H at the higher dose to attempt to achieve more comparable nasal titres between E21K and Y7H and also to enhance our chances of detecting airborne virus derived from minority variants.

**Fig 3:**
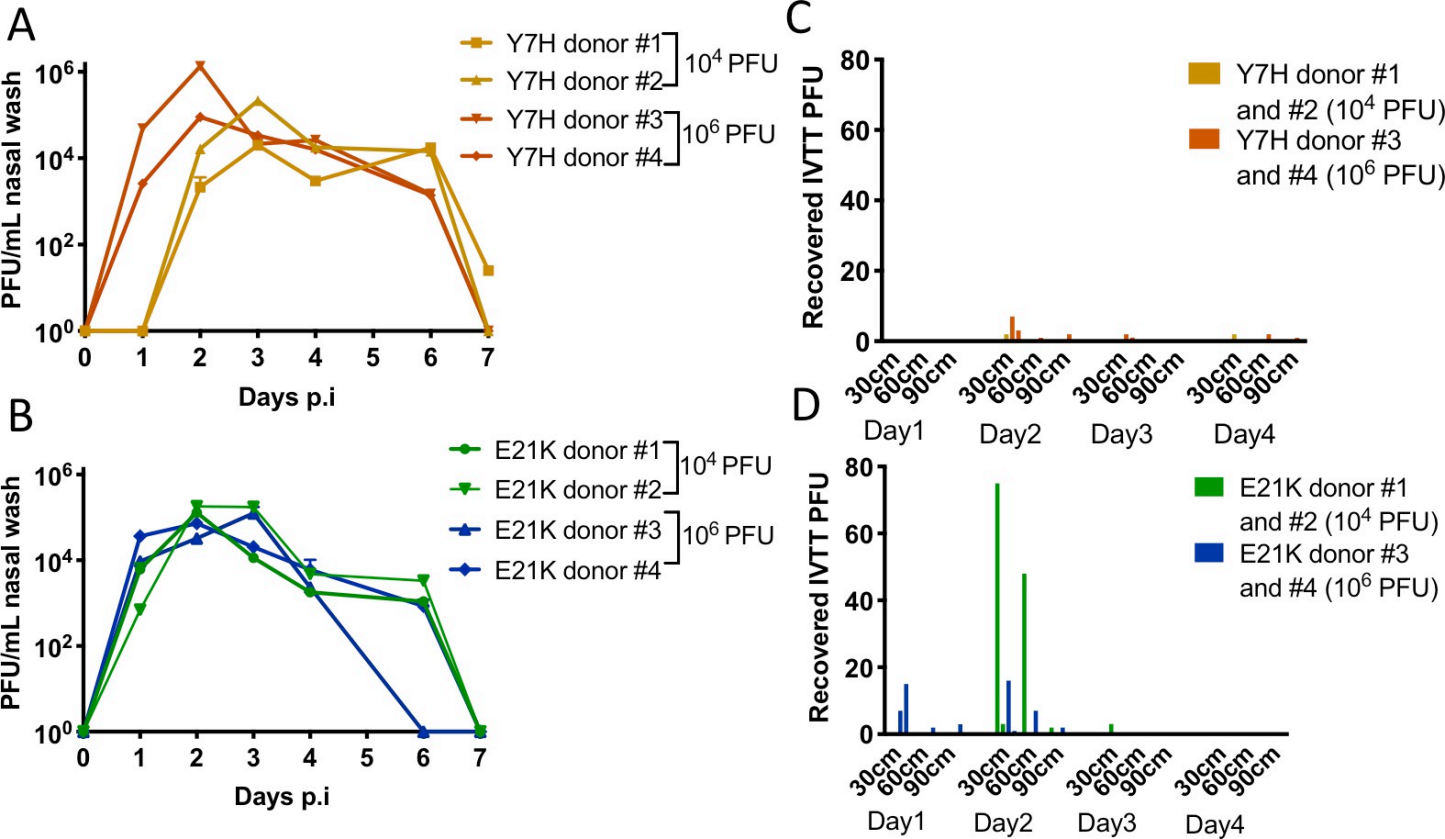
Infectious virus emitted from ferrets infected with pH1N1 virus mutants of varying pH stability. Four ferrets were infected with either Y7H (orange/red) or E21K (green/blue) viruses. In each group donors #1 and #2 were infected with 10^4^ PFU and donors #3 and #4 with 10^6^ PFU. Viral titres in nasal wash samples were quantified by plaque assay for (A) Y7H and (B) E21K infected ferrets. Virus emitted in airborne droplets was collected from (C) Y7H and (D) E21K infected ferrets in the IVTT for 10 minutes on days 1 to 4 and detected on culture plates placed at 30cm, 60cm and 90cm along the tunnel.

Levels and kinetics of viral replication in the ferret nose were not significantly different between E21K- and Y7H-infected ferrets by area under the curve (AUC) analysis, other than Y7H donor #3 that had an increased AUC and a higher peak nasal wash titre (Fig 3A and 3B). However, peak of shedding was delayed by one day in ferrets infected with 10^4^ PFU of Y7H compared to those infected at the higher dose or with E21K. Despite comparable levels of viral replication in the nasal tract, more plaques were recovered from air emitted by ferrets infected with the more stable E21K virus (total n = 184 plaques) than by the four ferrets infected with Y7H virus (total n = 23 plaques) (Fig 3C and 3D).

### Mutations that promote stability of haemagglutinin enhance virus survival in the air

Deep sequencing was performed on populations of virus obtained by nasal washing of infected ferrets on days 1–4 p.i and also on the viral inoculums (Eng09/E21K and Eng09/Y7H). Individual infectious virus plaques isolated from air emitted by infected ferrets using the IVTT were picked and Sanger sequencing of the viral HA and M genes was performed.

In samples isolated from E21K-infected ferrets, there were no new mutations detected in nasal virus compared to the viral inoculum (at >5% frequency by deep sequencing). In a selection of individual exhaled virus plaques collected on IVTT culture plates, there were no new mutations detected upon Sanger sequencing of HA or M genes. In contrast, in samples isolated from Y7H-infected ferrets, HA mutations were detected in both airborne and nasal virus that were not present in the inoculum virus.

**Fig 4:**
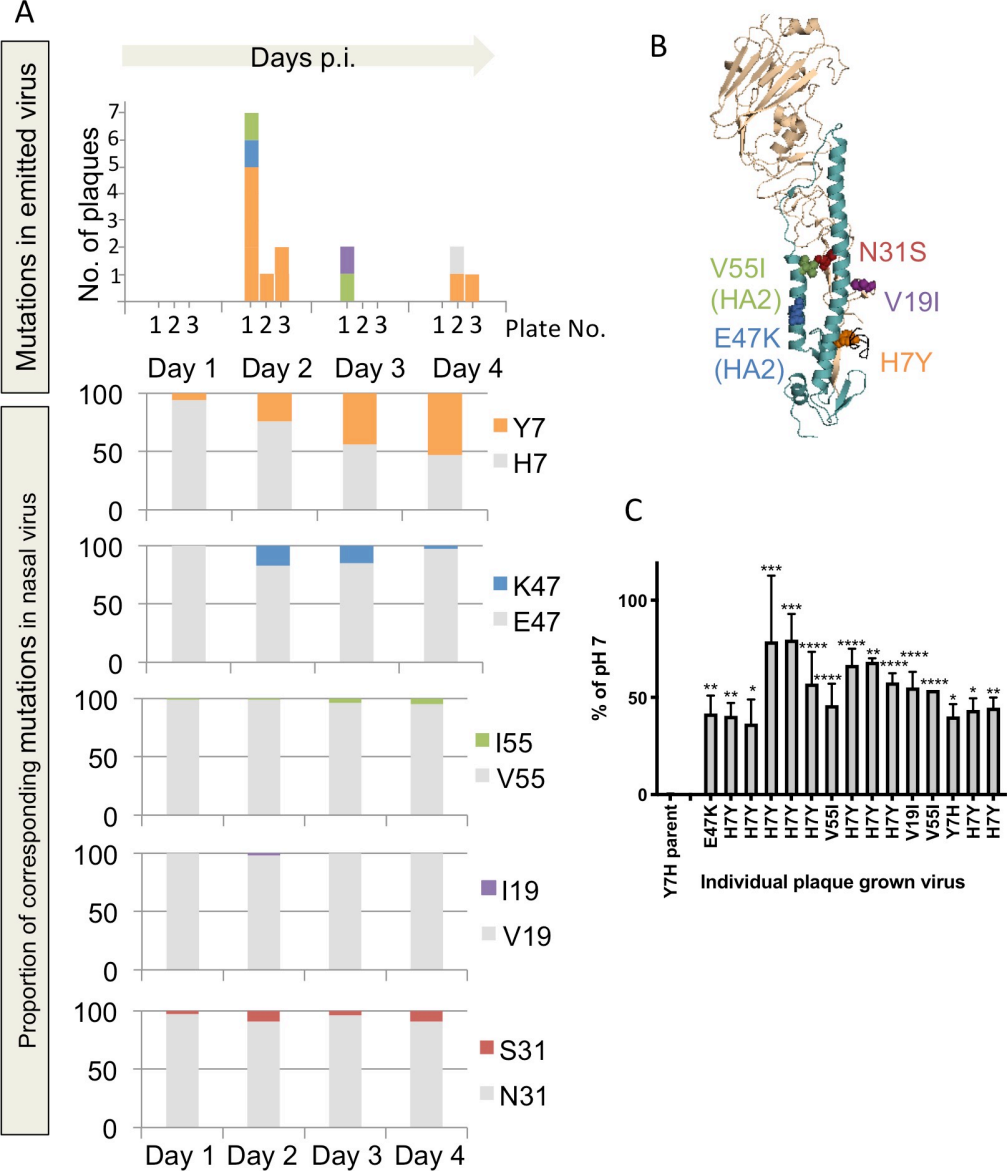
Mutations that promote stability of haemagglutinin enhance virus survival in the air. (A) Upper panel: Virus emitted by Y7H-infected ferret donor #3 on days 1 to 4 was collected as plaques picked from plates 1 (30cm), 2 (60cm) and 3 (90cm) of the IVTT and viral RNA extracted. The haemagglutinin (HA) gene was Sanger sequenced. HA mutations identified in viral plaques are represented by the colours orange H7Y, blue E47K-HA2, green V55I-HA2 and purple V19I. Lower panel: The proportion of corresponding HA mutations at positions 7 and 19 in HA1 and 47 and 55 in HA2 detected on days 1 to 4 in the nasal wash was determined by next-generation sequencing. An additional mutation detected in the nasal wash at >5% frequency but not in any picked plaques is also shown (N31S in red). In each of the bar graphs, the proportion of virus in nasal wash with sequence encoding the amino acid as in the parental virus (Y7H) is shown in grey. (B) HA mutations are modelled on a HA monomer using Pymol molecular visualization tool (PDB: 4jtv). H1 numbering using the mature HA sequence is used throughout[54]. HA1 is shaded light brown, HA2 is teal and the fusion peptide is black. (C) The acid stability of viruses in air emitted from donor #3 was tested by incubating virus propagated from IVTT plaques at low (pH 5.5) and neutral (pH 7) pH, in triplicate. The remaining infectivity detected at pH 5.5 is expressed relative to infectivity detected at pH 7. Each grey bar represents an individual virus propagated from a picked plaque with its HA mutation on the x-axis. Stability of parental virus Y7H is shown on the left of the panel. Error bars represent standard deviation of three independent experiments. One-way ANOVA with Tukey post-test was used to compare each plaque with the Y7H parent virus. *p<0.05, **p<0.01 ***p<0.001 ****p<0.0001.

The greatest number of virus plaques (n = 15) isolated from droplets emitted by a Y7H-infected ferret using the IVTT was from donor #3. In 14 out of 15 of these unique virus plaques, HA mutations were detected on Sanger sequencing,. These mutations included either a reversion at position 7 (H7Y) (in 10/15 plaques) or additional mutations located around the HA stem (V55I-HA2 in 2 plaques, E47K-HA2 in one plaque and V19I in one plaque) (Fig 4A and 4B). Interestingly, all of these mutations have previously either been detected during ferret transmission events, arisen during natural evolution of pH1N1, or been described to be stabilising to pH1N1 HA[10,31–33]. Phenotypically, all 15 virus plaques detected in the IVTT from donor #3 were found to have increased acid stability compared to the parent virus (Y7H) when tested for their ability to retain infectiousness at low pH (Fig 4C). Of note, one virus plaque emitted by donor #3 displayed a HA genotype unchanged from the parent virus (Y7H) yet displayed increased stability in the phenotypic assay. Whole genome sequencing of this virus subsequently identified an additional A202T mutation in the non-structural (NS) protein, which was also detected in the nasal wash of the donor animal at a frequency of 1.5%-5.8% and was not present in the inoculum. It may be that this mutation in NS is contributing, via an as yet unknown mechanism, to the virus’ ability to be exhaled and survive in airborne droplets[34].

In both donor #2 and donor #4, there was also evidence of survival of airborne virus with a pH stabilised phenotype and corresponding genotypic changes (S5 and S6 Figs). The HA mutations detected in these virus plaques included I57F (exhaled by donor #2) and V16I/T61P (exhaled by donor #4). These mutations are positioned in regions of HA that are expected to impact on pH stability (S5A, S5B, S6A and S6B Figs). The mutant viruses were found to be significantly more pH stable than the parent virus when tested for their ability to retain infectiousness at low pH (p<0.0001 by one-way ANOVA) (S5C and S6C Figs). Other plaques emitted by donors #2 and #4 that were isolated from the plate nearest the donor ferret did not show evidence of significantly increased stability. These were found to have retained the parental HA genotype (Y7H) or in the case of one plaque, had acquired only a T241I mutation located in the HA head, with no additional mutations detected in the other seven genes.

### Minority populations of virus in the nasal tract of Y7H-infected ferrets gave rise to the majority of airborne infectious virus detected in the IVTT

Interestingly, in the nasal wash of Y7H-infected ferrets, the mutations detected in exhaled plaques were present as minority populations including some existing as low frequency variants (<5% frequency) (Fig 4A and S5A and S6A Figs). These mutations were not detectable in the virus used to inoculate the animals. Data obtained by deep sequencing of the HA gene from the inoculum virus is shown in S3 Fig. The predominant mutation detected was a reversion at position 7 (H7Y) which arose rapidly by day 1 in the nasal wash of donors #3 and #4 and increased to 53% by day 4 (Fig 4 and S6 Fig). This reversion mutation was also seen in the day 4 nasal wash from donor #1 (S4 Fig). Donor #2 did not accumulate a position 7 reversion but instead showed different mutations in HA on day 3 (S5 Fig). For donor #3, the enrichment of the stable H7Y revertant in plaques recovered from air versus the representation of this genotype in nasal wash was statistically significant (p = 0.0003 by Fishers exact test).

All of the pH-stabilised plaques isolated from virus shed into the air carried mutations that were present in nasal washes, albeit detected as a minority population in several cases. Several other mutations were present at various frequencies in nasal wash of Y7H donor animals that were not present in the plaques isolated from air, perhaps because these mutations were not adequately stabilising to preserve virus viability in droplets or that due to sampling constraints these particular mutants were not detected as viral plaques. Taken together, our data show that virus mutants with more stable HA arose during replication in the ferret nasal tract and that increased pH stability can improve retention of infectivity in airborne droplets.

### The pH stable E21K virus has improved survival when nebulised into airborne droplets

**Fig 5:**
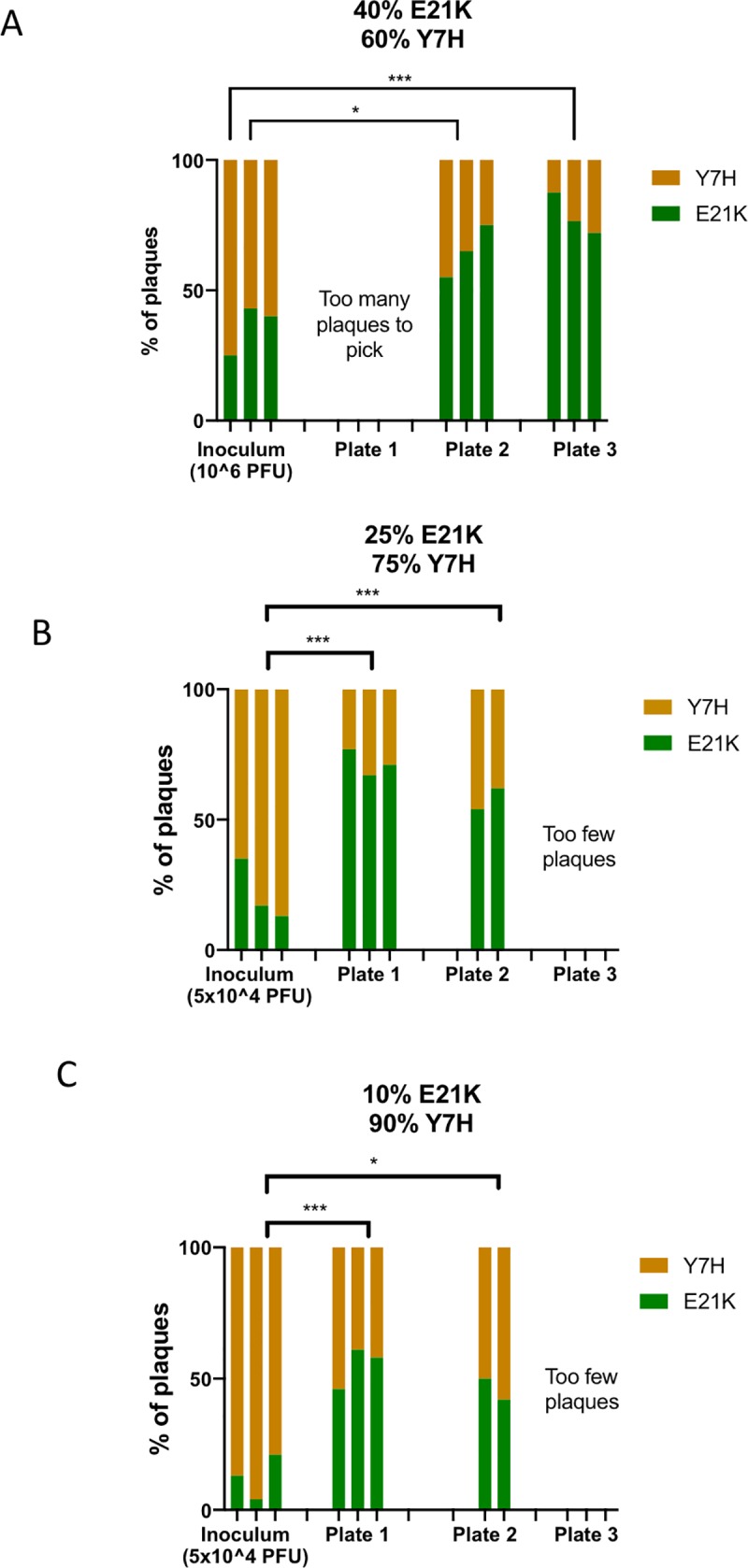
Virus with increased acid stability has improved survival when nebulised into droplets. (A) 40%:60% (B) 25%:75% and (C) 10%:90% mixtures of E21K and Y7H based on their infectivity by PFU/mL were nebulised into the IVTT in three independent experiments. Input viral titres were 10^6^ PFU in (A) and 5x10^4^ PFU in (B) and (C). Viral plaques that formed on IVTT culture plates 1 (at 30cm), 2 (at 60cm) and 3 (at 90cm) and a sample of plaques from the inoculum were picked and Sanger sequencing of the haemagglutinin gene carried out. Where the distribution of plaques was such that too few (n = <5) or too many plaques were collected to enable purification and/or analysis is indicated. Fisher’s exact test was used to compare the proportions of E21K/Y7H genotype detected on each plate. *p≤0.05, **p≤0.01, ***p≤0.001.

To validate our findings in ferrets that demonstrate a survival advantage in droplets conferred by increased HA stability, we performed further studies with nebulised virus. A 40:60% mixture of E21K and Y7H viruses (total 10^6^ PFU) was nebulised into the IVTT (Fig 5A). Virus plaques collected on plates 2 (n = 70) and 3 (n = 75) were picked and Sanger sequencing of the HA gene performed. There were too many plaques on plate 1 to enable individual plaques to be picked. The proportion of plaques with the pH stable E21K genotype was found to be significantly increased on plate 2 (65%, p = 0.024) and plate 3 (79%, p = 0.0002) compared to the inoculum (36%), suggesting the virus with more stable HA was better able to retain infectivity when nebulised into droplets.

To further confirm this finding, we repeated the experiment using a mixture containing a lower titre of inoculum virus to enable individual plaques to be isolated from plate 1, which is more representative of the distribution of plaques emitted in ferret exhaled breath. A 25:75% mixture of E21K and Y7H (total 5x10^4^ PFU) was nebulised into the IVTT (Fig 5B). Virus plaques collected on plates 1 (n = 70) and 2 (n = 26) were picked and Sanger sequencing of the HA gene performed. There were too few plaques collected on plate 3 (<5 plaques). The proportion of plaques with the pH stable E21K genotype was again found to be significantly increased on plate 1 (72%, p<0.0001) and plate 2 (58%, p = 0.001) compared to the proportion in the inoculum (22%). Finally, we attempted the same experiment using an even lower proportion of E21K to examine if the observation would hold true even when the stable virus was present in a minority. A 10:90% mixture of E21K and Y7H (total 5x10^4^ PFU) was nebulised into the IVTT (Fig 5C). Virus plaques collected on plates 1 (n = 71)) and 2 (n = 22) were picked and Sanger sequencing of the HA gene performed. There were too few plaques collected on plate 3 (<5 plaques). The proportion of plaques with the pH stable E21K genotype was again significantly increased on plate 1 (55%, p<0.0001) and plate 2 (46%, p = 0.02) compared to the proportion in the inoculum (13%) supporting the hypothesis that HA stability can enhance the ability of virus to remain infectious in airborne droplets.

## Discussion

The kinetics of infection in an influenza-infected animal or human is typically monitored by virus quantification from a nasal wash or nasal swab. However, these methods might artificially collect virus that would never be naturally released, or dilute inhibitory factors that otherwise negatively impact the survival of virus released into airborne droplets or aerosols. For virus to successfully transmit through the air, it must be exhaled from the donor in sufficient quantities and retain infectiousness as it travels between hosts. Compared to nasal wash sampling from infected ferrets, far less infectious virus has been detected by air sampling in several studies using different methodologies[3,13,17]. Whilst difficulties capturing viable airborne viruses are likely contributing to this discrepancy to an extent, it also highlights gaps in understanding about influenza transmission between hosts. How much contagious virus is naturally released into the air? How representative are nasal wash titres for contagious virus that is naturally released? What viral, host and environmental factors affect the ability of virus to be released and survive in airborne droplets? Our study describing a unique method for collection of airborne infectious virus aims to improve understanding of between-host transmission and is the first report isolating and characterising the genotype and phenotype of viable viruses collected from airborne droplets.

Using our technique of direct viral plaque isolation, we show that ferrets infected with an airborne transmissible pH1N1 virus exhaled a peak of infectious virus early in infection. The quantity of infectious virus detected by IVTT sampling decreased with time post infection. This pattern correlates with previous reports of an early peak of airborne contagiousness in ferrets, prior to the onset of fever and clinical symptoms[2,5,26]. In these studies, contagiousness was found to decrease as infection progressed and clinical symptoms such as coughing, sneezing and nasal congestion became more apparent. We did not observe any coughing or sneezing by virus-infected ferrets whilst they were sampled in the IVTT apparatus early after infection. Previous studies that have focussed on measuring exhaled viral RNA showed no correlation between airborne RNA levels and contagiousness, actually detecting more airborne viral RNA during times of reduced contagiousness[2] and for several days after infection[2,3,5]. The reason that virus is not transmitted by the respiratory route late in infection despite high titres of virus in ferret nasal washes and viral RNA detectable in exhaled air, warrants further investigation. Perhaps other as yet uncharacterised inhibitory host factors limit survival of exhaled virus later in the course infection, for example production of innate immune mediators or a change in the local environment of the upper respiratory tract such as pH, viscosity, or mucous production, resulting in altered composition of respiratory secretions. Future studies might seek to analyse the composition and physicochemical properties of ferret respiratory secretions over the course of infection.

We calculated that ferrets could emit as much as 138 PFU/minute of infectious pH1N1 virus, which is a 1–2 order of magnitude higher than previous estimates. Using an impactor to collect exhaled particles from ferrets over 30 minutes, Gustin *et al*.[3,23] calculated a rate of <4 PFU/minute. The largest quantity of virus they directly collected from a single ferret in 30 minutes was 11 PFU[23], in contrast to 327 PFU in one third of the sampling time using our technique. Therefore, despite the limited sampling time of 10 minutes and potential losses from particle deposition on the outer walls of the chamber, plaque collection is a more sensitive method for isolating airborne infectious virus than impaction or biosampling devices previously described[35]. Others have attempted collection of viable virus exhaled by humans but with minimal success, requiring artificial breathing manoeuvres and secondary culture steps that abrogate virus quantification[14,17,18,36,37]. Yan *et al*.[13] detected virus in small aerosols (<5μm) exhaled naturally by human subjects with a maximum of 37 focus forming units (FFU)/minute from one subject. One caveat of this work is that quantification using FFU that assesses virus in a single round of replication, rather than a multicycle analysis such as PFU, would incorrectly score incomplete viral particles as viable. In addition, droplets >5μm, which may have contained significant amounts of viable virus, were excluded from analysis due to sampling constraints related to inefficiency of the impactor used for virus collection.

Characterising individual virus plaques isolated in the IVTT enabled us to demonstrate a role for stability of influenza HA for retention of viability in airborne droplets. The more stable “human virus-like” E21K mutant was better able to retain infectivity when nebulised into droplets than the less stable “avian virus-like” Y7H mutant. Infectious virus recovered from droplets exhaled by ferrets inoculated with Y7H virus contained mutations that conferred restabilisation of HA. This concurs with the findings of Russier *et al*.[10] who detected a single stabilised mutant (HA H7Y+R106K) in 1 out of 4 sentinel ferrets exposed to donors infected with pH1N1/Y7H in a traditional ferret-to-ferret transmission experiment. Importantly, after virus enters and replicates in a sentinel animal, further selection limits the information that can be gained about the transmitted virus. For example, it is not possible to delineate whether selection of a pH-stable mutant has occurred during replication in the donor, at the point of release from the donor, during travel in the air between animals, or during infection of the sentinel animal. Using the IVTT to sample virus from exhaled droplets has allowed us to capture information about several stabilised mutants released into the air without the need for virus to infect and replicate in an onward host. Strikingly, we observed that whilst only minority populations of pH stable viruses were detected in the donor ferret nose, it is predominantly these rare mutants that are detected in the air.

Within airborne droplets, the viral envelope must withstand conditions quite distinct from the environment of the URT. Evaporation of water occurs rapidly (in < 1 second) after droplets are released from the URT[38]. Others have suggested that an increased concentration of salts and insoluble solutes in droplet nuclei[38,39], osmotic forces[40], or inactivation of virus accumulating at the surface of droplets due to surface tension might explain infectivity losses[41]. As a consequence of water evaporation, the concentration of free H+ ions in a droplet increases and lowers the pH. For example, Yang and Marr[41] calculated that at 60% relative humidity, similar to conditions during our IVTT experiments, a droplet will shrink to 0.17 of its initial diameter, reducing pH by 2.3 units. This effect might be compounded at the air/water interface at the surface of droplets[42]. Human nasal mucosa is mildly acidic (ranging from 5.3 to 7.0)[11] so a decrease in pH in droplets consisting of fluid derived from the nasal mucosa of up to 2.3 units would fall within the pH range that can trigger HA unfolding. Moreover, inhibitory substances incorporated into droplets formed from respiratory secretions may become more concentrated in droplet nuclei, which could contribute to infectivity losses. Of note, a recent study showed a correlation between loss of infectivity in aged aerosols and poor transmission[43].

As with all methods employed to isolate influenza virus from the air, there are limitations to the design of the IVTT that must be considered. The IVTT is uniquely designed as a straightforward and sensitive method to sample infectious virus from airborne droplets depositing on cell culture plates but the ability to collect infectious virus from small aerosols is less certain. Interestingly, emerging data demonstrates that droplets play a central role in transmissibility between ferrets. Zhou *et al*.[2] demonstrated that a large proportion of viral RNA exhaled by ferrets exists in particles >4.7μm in size (no maximum size cut off) and that presence of larger droplet sizes increased airborne transmissibility. Airborne transmission did not occur when only aerosols <1.5μm were present, suggesting that fine aerosols do not contain significant amounts of contagious virus, despite representing 76.8% of particles released by experimentally-infected ferrets. Gustin *et al*.^14^ also detected a five times higher amount of viable virus in particles >4.7μm despite the most frequent size of exhaled particle being <1μm. Our technique was unsuccessful in collecting viral genome copies emitted by pH1N1-infected ferrets, likely because quantities collected in 10 minutes are below the limit of detection by RT-qPCR. To overcome this limitation, a longer ferret exposure time would be necessary to increase virus in the IVTT to a measureable quantity. However, because ferrets are introduced into the chamber while fully conscious (to allow for natural breathing), a maximum breath collection of 10 minutes is applied for animal welfare reasons. We did note ferret-to-ferret variability in the quantities of airborne infectious virus detected, which has also been observed in previous studies on ferrets[3] and humans[13]. It is possible that this variability represents differences in individual ferret physiology or in how the timing of the 10-minute IVT sampling period relates to virus-host kinetics in the individual.

Although ferrets are considered the best available model for influenza transmission, these studies will never truly represent human infection. The duration of human contagiousness following primary infection with influenza and the relative contribution of different routes of transmission and particle sizes remain critical unknowns in the field. Employing the IVTT technique to sample from naturally or experimentally infected humans might provide valuable information on contagious virus shedding. In ferrets, others have shown that infection with airborne transmissible viruses result in more viable exhaled virus than those that do not transmit[23] and observed correlation between infectivity in aged aerosols and transmissibility[43]. Whether pH stability confers an airborne transmission advantage for all influenza viral subtypes including pre-pandemic and zoonotic viruses with pandemic potential will require further investigation.

Overall, the novel methodology and findings described here highlight the fate of infectious virus outside the respiratory tract as an important parameter for understanding influenza transmission. Direct measurements of airborne infectious virus and characterisation of their genotype and phenotype can significantly enhance the utility and quality of information obtained from ferret transmission studies.

## Materials and methods

### Cells

Madin-Darby Canine Kidney (MDCK) cells (ATCC) were maintained in Dulbecco’s modified Eagle’s Medium (DMEM) (Gibco-Life technologies) supplemented with 10% foetal bovine serum (FBS) (Labtech International), 1% penicillin and streptomycin (Gibco- Life technologies) and 1% nonessential amino acids (Sigma-Aldrich) at 37°C with 5% CO_2_.

### Viruses

The prototypic 2009 pandemic H1N1 (pH1N1) virus A/England/195/2009 (Eng09) was derived using reverse genetics by *de novo* synthesis (GeneArt) from published sequence data. E21K and Y7H point mutations were introduced into a pPol1 plasmid containing the HA of Eng09 using QuikChange site-directed mutagenesis kit (Stratagene) according to manufacturer’s instructions. Viruses were generated using a 12 plasmid reverse genetics system as previously described[44] and propagated in flasks of confluent MDCK cells in the presence of DMEM (1% Penicillin/Streptomycin) and 1μg/ml TPCK treated trypsin (Worthington Biosciences). Virus stock titres were determined by plaque assay on MDCK cells.

### Animal studies

Animal studies were conducted as previously described[45]. Female ferrets (20–24 weeks old) weighing 500–1000g were used. Body weight was measured daily. After acclimatization, ferrets were lightly anaesthetized with ketamine (22 mg/kg) and xylazine (0.9 mg/kg) and inoculated intranasally with Eng09/wild type (10^4^ PFU), Eng09/E21K or Eng09/Y7H virus (10^4^ or 10^6^ PFU as stated) diluted in phosphate buffered saline (PBS) (0.1 ml per nostril). All animals were nasal washed daily, while conscious, by instilling 2 ml PBS into the nostrils and the expectorate collected in modified 250 ml centrifuge tubes. Infectious virus was titrated immediately by plaque assay of the nasal wash on MDCK cells.

### The influenza virus transmission tunnel

The influenza virus transmission tunnel (IVTT) was designed in house and manufactured by EMMS systems (Hampshire, UK). The system consists of a bias flow pump (EMMS) connected to a 37.5cm (height) x 25cm (diameter) ferret chamber. The ferret chamber is connected to the IVTT (a 100cm (length) x 18cm (width) x 9cm (height) half-cylindrical clear acrylic exposure tunnel) by a 1.5 cm (diameter) aperture allowing free passage of air. Sentinel cell plates are centred at 30, 60 and 90 cm from the tunnel opening and can be accessed via drawers from the side of the IVTT. Air is channelled from the exit port of the IVTT toward a downflow bench or microbiological safety cabinet filter to provide a low level draw. For air sampling, an SKC Biosampler (SKC Inc.) is connected to the exit port of the IVTT, with air pulled through at a rate of 12.5L/min. The ferret chamber is replaced by a 10cm (height) x 9cm (diameter) nebulisation chamber (EMMS) attached to an Aerogen Pro nebuliser (Aerogen), which generates droplets with a volume mean diameter (VMD) of 4 to 6μm, for *in vitro* experiments. All experiments were conducted with minimal-to-no UV light exposure.

### In vivo IVTT experiments

Infected ferrets were placed into the ferret chamber for 10 minutes per exposure. Ferrets were not sedated during the exposure period and therefore, for well being, exposure was restricted to 10 minutes. Airflow of 7.5L/minute was introduced using the bias flow pump via three ports into the ferret chamber (2.5L/minute into each port), which connects to the IVTT via a 1.5cm diameter opening during the exposure period. Analysis of the impact of air flowing through a 1.5cm opening at 7.5L/minute found no suggestion that particle distribution would be altered or particle breakup would occur based on calculations of the Ohnesgorge and Weber numbers. Sentinel 6-well plates containing a confluent monolayer of MDCK cells overlaid with 0.5mL overlay medium were added to the tunnel for the duration of the exposure period. Overlay medium contained a final concentration of 1xMEM, 0.21% bovine serum albumin (BSA) V, 1mM L-glutamate, 0.15% sodium bicarbonate, 10mM HEPES, 1x penicillin-streptomycin (all from Gibco) and 0.01% dextran DEAE (Sigma). The plates were incubated for one hour at 37°C, 5% CO_2_ prior to the addition of a semi-solid agarose overlay medium containing 2.5μg/mL of amphotericin B and 1μg/mL TPCK treated trypsin and incubated for a further 3 days to allow for formation of viral plaques. Between exposures for each donor ferret, sentinel cells were replaced and the collection chamber and cell tunnel cleaned to remove any surface deposited virus. Ferrets were nasal washed after IVTT exposures had been carried out. Temperature and humidity were monitored throughout experiments and observed to be 21+/-1°C and 50+/-10% respectively. The IVTT was disinfected after use with a 1% virucidal disinfectant solution. All experiments were conducted at containment level 2.

### In vitro IVTT experiments

Viruses (and/or plasmid DNA) under study were diluted in PBS and nebulised using the Aerogen Pro nebuliser into the nebuliser chamber attached to the IVT tunnel. Airflow at a rate of 1L/minute was introduced via two ports into the nebuliser chamber, which connects to the IVTT. Sentinel MDCK cells overlaid with 0.5mL overlay medium were exposed for 10 minutes per nebulisation and after exposure the plates were incubated for one hour at 37°C, 5% CO_2_ prior to the addition of semi-solid agarose overlay medium containing 1μg/mL TPCK treated trypsin and incubated for a further 3 days to allow formation of viral plaques. Viral RNA or tracer DNA were collected from1120μL PBS supplemented with 0.375% BSA-V placed in the central space between the wells of the three 6-well IVTT culture plates. Where air sampling was undertaken, after the 10 minute collection window, air remaining in the IVTT tunnel was pulled through a SKC Biosampler (SKC Inc.) at a rate of 12.5L/minute (calibrated as per manufacturer’s instructions), for a further 10 minutes. Air samples were collected into 15mL PBS and quantified immediately by plaque assay on MDCK cells. Temperature and humidity were monitored throughout experiments and were 21+/-1°C and 50+/-10% respectively. All experiments were performed with the IVTT placed within a class II biological safety cabinet at containment level 2. The IVTT was disinfected after use with a 1% virucidal disinfectant solution.

### Real-time quantitative PCR

Real-time quantitative PCR (RT-qPCR) was used to quantify the amount of plasmid DNA or viral RNA deposited on each culture plate and from an air sample collected using a SKC Biosampler. Viral RNA was extracted using the QIAamp viral RNA mini it (Qiagen) from 140μL samples and eluted into 40μL water. RT-qPCR of the viral M gene or DNA plasmid was performed using AgPath-ID One-Step RT-PCR Reagents (Thermo Fisher Scientific) with specific primers and probes[46]. Primer sequences are available on request. Data was analysed on the QuantStudio 7 Flex Real-Time PCR System. The plasmid copy number was calculated against a standard curve generated from serial 10-fold dilutions from 2x10^8^ to 2x10^0^ copies/μL.

### Conversion calculations of virus detection in the IVTT

Viral plaque counts were taken from 6-well plates centred at 30cm (plate 1), 60cm (plate 2) and 90cm (plate 3). Plaque counts from the 6 wells were divided by the measured surface area to obtain an estimate for the viral plaques per square metre, or plaque density, at that recorded point. As the results were taken in triplicate, this then allowed for a set of upper, lower and mean values at each site. The decay in plaque numbers between subsequent sites was somewhere between linear and exponential across all ferrets. An exponential or linear regression fit was adopted accordingly to model the viral plaque densities, *N*, at a distance *x*. By integrating the plaque density over definite limits we formed an estimate for the total number of plaques, *P*, within a given area, *A*:

$$P=\int_{A}N\;dA=L_{z}\int_{x_{1}}^{x_{2}}N\;dx$$

where *x*_*1*_ and *x*_*2*_ represent axial distances along the tube, and *L*_*z*_ is the spanwise width.

### Modelling droplet trajectories in the IVTT

Airflow into the IVTT was modelled as a laminar round jet[47], and a Lagrangian particle-tracking algorithm adopted to compute the droplet trajectories[48]. The forces acting on the droplets were taken to be drag and gravity, which are the dominant forces for aerosol particles in the micrometre range[49]. The change in droplet size and temperature resulting from evaporation of water to the surrounding air was also taken into account, based on previous modelling approaches for soluble aerosol particles[50,51]. Droplets were released from the inlet tube into the IVTT at random locations drawn from a uniform distribution, and tracked until they either deposited or exited through the outlet.

### Viral plaque sequencing

Viral plaques were picked using a Gilson pipette and propagated on MDCK cells. Viral RNA was isolated using QIAamp viral RNA mini kit (Qiagen) and reverse transcribed using SuperScript III reverse transcriptase (Invitrogen) with random hexamers. The HA or M gene was amplified using KOD polymerase (Thermo Fisher Scientific) with specific primers (sequences available on request) and Sanger sequenced.

### Next-generation sequencing

Whole genome sequencing of influenza A was performed using total RNA extracted directly from 150μL of ferret nasal wash samples using Easymag, according to manufacturer instructions. Multisegment reverse transcription-PCR (M-RTPCR)[52] was used to amplify influenza virus-specific segments. Reverse-transcription and amplification was performed using One-step RT-PCR system with Superscript III and Platinum Taq HiFi polymerase (Life Technologies). The M-RTPCR products were cleaned, diluted to required concentration and submitted for Nextera library preparation for Illumina short-read sequencing with a MiSeq instrument. Sequence data generated using the Illumina MiSeq was processed using BWA-MEM to map the reads to appropriate reference sequences. Samtools[53] was used to post-process the reference assembly and an in-house C++ program (QuasiBam) used to quality filter, trim and generate two outputs: a consensus sequence for the influenza genomes and a table with the frequency of each nucleotide and depth at every position. Positions in the consensus genome showing greater than 20% variance were assigned an ambiguity code. Variant calling required a minimum of 10 reads and a minimum read frequency cutoff of 5% was set.

### Acid inactivation assay

Each virus was mixed with MES buffer (100mM MES, 150mM NaCl, 0.9mM CaCl_2_, 0.5mM MgCl_2_) adjusted to pH 5.5 or 7.0 in triplicate and incubated for 15 minutes at 37°C. The buffer was inactivated with a 10-fold dilution in DMEM and samples titrated by plaque assay on MDCK cells.

### Statistical analysis

Statistical analysis was performed using Prism 7 software (GraphPad Software, San Diego, CA). A p value <0.05 was considered significant.

### Ethics statement

All work was approved by the local genetic manipulation (GM) safety committee of Imperial College London, St. Mary’s Campus (centre number GM77), and the Health and Safety Executive of the United Kingdom. Animal work was performed under a United Kingdom Home Office License, PPL 70/7501 in accordance with the approved guidelines, under the Animals (Scientific Procedures) Act 1986 (ASPA).

## Supporting information

S1 Fig Distribution of infectious virus, viral RNA and tracer DNA nebulized into the IVTT. 2x10^7^ PFU of Eng/09 virus and 1x10^9^ copies of plasmid DNA diluted in 100 μL PBS were nebulised into the IVTT simultaneously and cell culture plates exposed for 10 minutes, in triplicate. Infectious virus was collected as plaque forming units (PFU) on MDCK cell culture plates (white bars). Plasmid DNA and viral RNA were collected into 1120μL PBS supplemented with 0.375% BSA-V place in the central space of the 6 well plates and quantified by real-time quantitative PCR (black and grey bars). After the 10 minute collection window, air was sampled into 15mL PBS with a SKC Biosampler connected to the end of the IVTT for a further 10 minutes and subjected immediately to plaque assay for infectious virus and real-time quantitative PCR for DNA and viral RNA. Error bars show standard deviation of three independent experiments.
(PDF)

S2 Fig Distribution of infectious virus isolated in the IVTT from pH1N1-infected ferrets. Six ferrets were intranasally inoculated with 10^4^ PFU in 0.1mL PBS pH1N1 Eng/09 virus. On day 2 post inoculation, the number of exhaled infectious viral plaques detected on IVTT plate 1 (30cm, black), 2 (60cm, grey) and 3 (90cm, white) for each of the six donor ferrets was counted.
(PDF)

S3 Fig Next-generation sequencing of Y7H virus inoculum. The Y7H virus inoculum was interrogated for the presence of any low frequency mutations. Data on the amino acid present in the positions at which mutations were detected in IVTT plaques exhaled by Y7H-infected ferrets is shown.
(PDF)

S4 Fig Sequencing of airborne and nasal virus isolated from Y7H-infected donor #1. (A) Upper panel: No virus was detected in the IVTT from Y7H-infected ferret donor #1 on days 1 to 4. Lower panel: Nasal wash from days 1 to 4 was next-generation sequenced and variants present at >5% frequency are shown in the bar graph: H7Y orange, R54G blue, S289N green and D19N in HA2 purple. In each of the bar graphs, the proportion of virus in nasal wash with sequence encoding the amino acid as in the parental virus (Y7H) is shown in grey. (B) HA mutations are modelled on a HA monomer using Pymol molecular visualization tool (PDB: 4jtv). H1 numbering using the mature HA sequence is used throughout[54]. HA1 is shaded light brown, HA2 is teal and the fusion peptide is black.
(PDF)

S5 Fig Sequencing of airborne and nasal virus isolated from Y7H-infected donor #2. (A) Upper panel: Virus emitted by Y7H-infected ferret donor #2 on days 1 to 4 was collected as plaques picked from plates 1 (30cm), 2 (60cm) and 3 (90cm) of the IVTT and viral RNA extracted. The haemagglutinin (HA) gene was Sanger sequenced. HA mutations identified in viral plaques are represented by the colours orange V19I, blue I57F, green T241I and purple L98M-HA2. Lower panel: The proportion of corresponding HA mutations on days 1 to 4 in the nasal wash was determined by next-generation sequencing. In each of the bar graphs, the proportion of virus in nasal wash with sequence encoding the amino acid as in the parental virus (Y7H) is shown in grey. (B) HA mutations are modelled on a HA monomer using Pymol molecular visualization tool (PDB: 4jtv). H1 numbering using the mature HA sequence is used throughout[54]. HA1 is shaded light brown, HA2 is teal and the fusion peptide is black. (C) The acid stability of viruses in air emitted from donor #2 was tested by incubating virus propagated from IVTT plaques at low (pH 5.5) and neutral (pH 7) pH, in triplicate. The remaining infectivity detected at pH 5.5 is expressed relative to infectivity detected at pH 7. Each grey bar represents an individual virus propagated from a picked plaque with its HA mutation on the x-axis. Stability of parental virus Y7H is shown on the left of the panel. Error bars represent standard deviation of three independent experiments. One-way ANOVA with Tukey post-test was used to compare each plaque with the Y7H parent virus. *p<0.05, **p<0.01 ***p<0.001 ****p<0.0001.
(PDF)

S6 Fig Sequencing of airborne and nasal virus isolated from Y7H-infected donor #4. (A) Upper panel: Virus emitted by Y7H-infected ferret donor #4 on days 1 to 4 was collected as picked plaques from plates 1 (30cm), 2 (60cm) and 3 (90cm) of the IVTT and viral RNA extracted. The haemagglutinin (HA) gene was Sanger sequenced. HA mutations identified in viral plaques are represented by the colours orange H7Y, blue V16I, green T61P in HA2. Lower panel: The proportion of corresponding HA mutations at positions 7 and 16 in HA1 and 61 in HA2 detected on days 1 to 4 in the nasal wash was determined by next-generation sequencing. No additional mutations were detected in the nasal wash at >5% frequency. In each of the bar graphs, the proportion of virus in nasal wash with sequence encoding the amino acid as in the parental virus (Y7H) is shown in grey. (B) HA mutations are modelled on a HA monomer using Pymol molecular visualization tool (PDB: 4jtv). H1 numbering using the mature HA sequence is used throughout[54]. HA1 is shaded light brown, HA2 is teal and the fusion peptide is black. (C) The acid stability of viruses in air emitted from donor #4 was tested by incubating virus propagated from IVTT plaques at low (pH 5.5) and neutral (pH 7) pH, in triplicate. The remaining infectivity detected at pH 5.5 is expressed relative to infectivity detected at pH 7. Each grey bar represents an individual virus propagated from a picked plaque with its HA mutation on the x-axis. Stability of parental virus Y7H is shown on the left of the panel. Error bars represent standard deviation of three independent experiments. One-way ANOVA with Tukey post-test was used to compare each plaque with the Y7H parent virus. *p<0.05, **p<0.01 ***p<0.001 ****p<0.0001.
(PDF)
