# Supplementary figures and images for: Characterising viable virus from air exhaled by H1N1 influenza-infected ferrets reveals the importance of haemagglutinin stability for airborne infectivity
Source: PLoS Pathog. 2020 Feb 25;16(2):e1008362. doi: 10.1371/journal.ppat.1008362 (PMC7059951; doi:10.1371/journal.ppat.1008362)

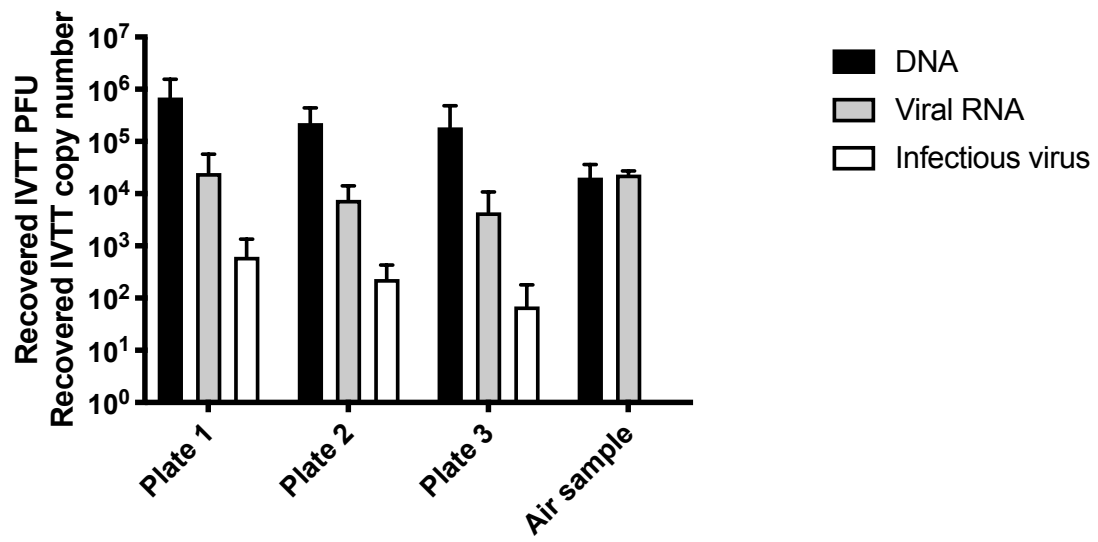

Fig S1

Supplement: S1 Fig — 2x10^7^ PFU of Eng/09 virus and 1x10^9^ copies of plasmid DNA diluted in 100 μL PBS were nebulised into the IVTT simultaneously and cell culture plates exposed for 10 minutes, in triplicate. Infectious virus was collected as plaque forming units (PFU) on MDCK cell culture plates (white bars). Plasmid DNA and viral RNA were collected into 1120μL PBS supplemented with 0.375% BSA-V place in the central space of the 6 well plates and quantified by real-time quantitative PCR (black and grey bars). After the 10 minute collection window, air was sampled into 15mL PBS with a SKC Biosampler connected to the end of the IVTT for a further 10 minutes and subjected immediately to plaque assay for infectious virus and real-time quantitative PCR for DNA and viral RNA. Error bars show standard deviation of three independent experiments. (PDF) [file ppat.1008362.s001.pdf]

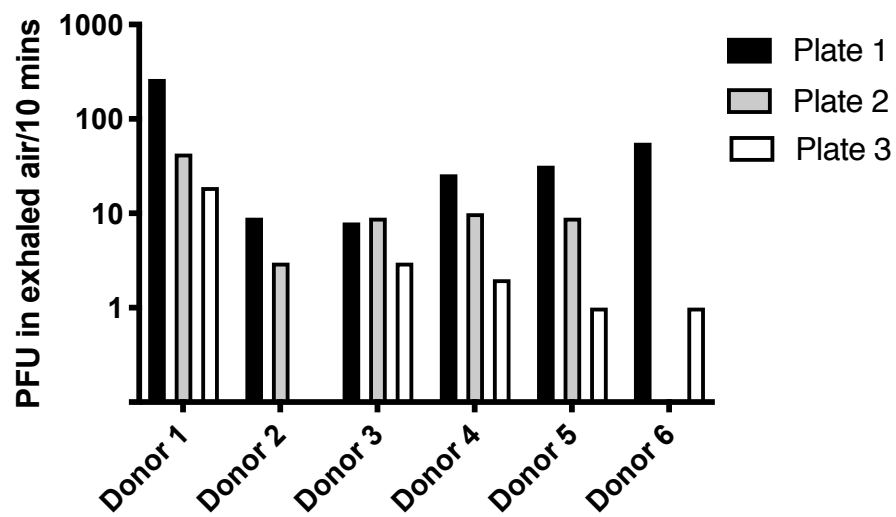

Fig S2

Supplement: S2 Fig — Six ferrets were intranasally inoculated with 104 PFU in 0.1mL PBS pH1N1 Eng/09 virus. On day 2 post inoculation, the number of exhaled infectious viral plaques detected on IVTT plate 1 (30cm, black), 2 (60cm, grey) and 3 (90cm, white) for each of the six donor ferrets was counted. (PDF) [file ppat.1008362.s002.pdf]

A

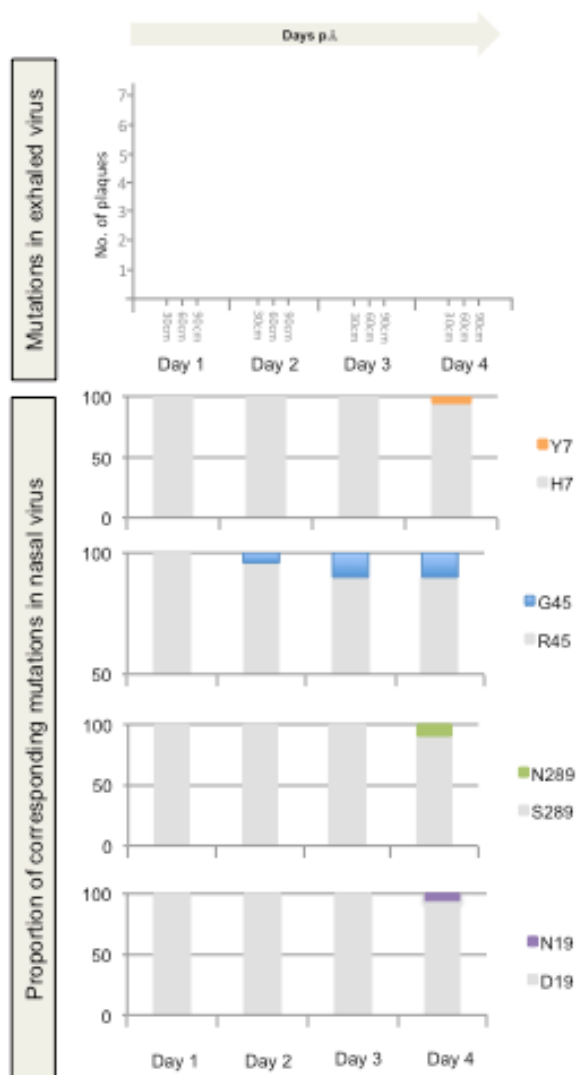

B

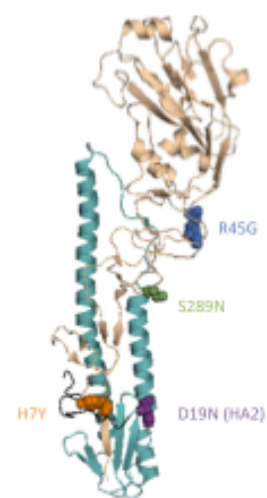

Fig S4

Supplement: S4 Fig — (A) Upper panel: No virus was detected in the IVTT from Y7H-infected ferret donor #1 on days 1 to 4. Lower panel: Nasal wash from days 1 to 4 was next-generation sequenced and variants present at >5% frequency are shown in the bar graph: H7Y orange, R54G blue, S289N green and D19N in HA2 purple. In each of the bar graphs, the proportion of virus in nasal wash with sequence encoding the amino acid as in the parental virus (Y7H) is shown in grey. (B) HA mutations are modelled on a HA monomer using Pymol molecular visualization tool (PDB: 4jtv). H1 numbering using the mature HA sequence is used throughout[54]. HA1 is shaded light brown, HA2 is teal and the fusion peptide is black. (PDF) [file ppat.1008362.s004.pdf]

A

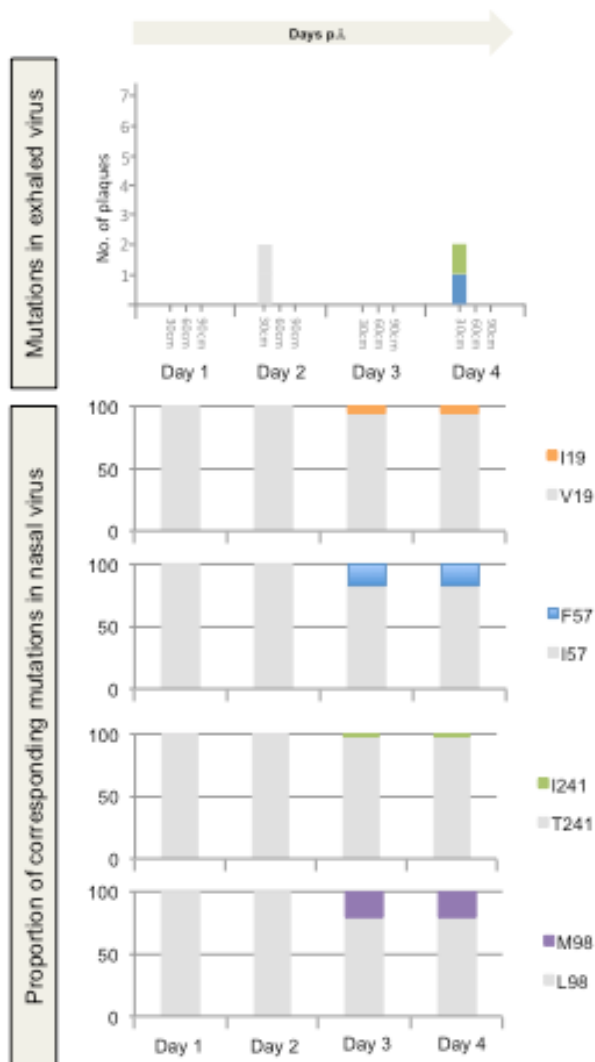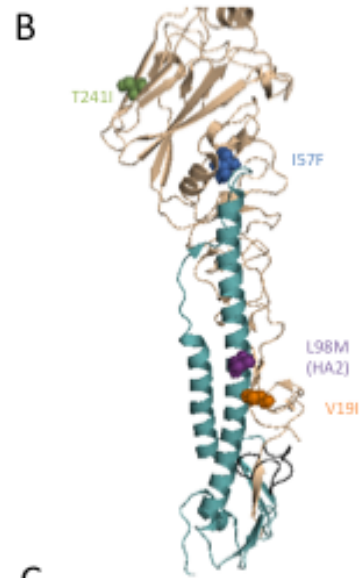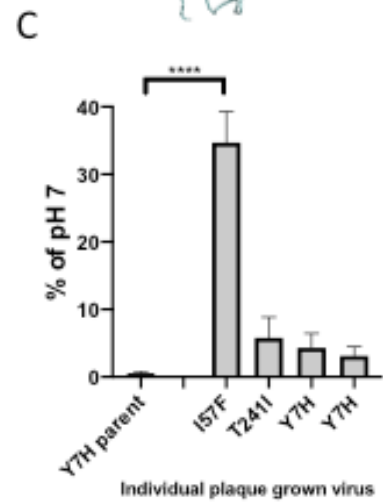

Fig S5

Supplement: S5 Fig — (A) Upper panel: Virus emitted by Y7H-infected ferret donor #2 on days 1 to 4 was collected as plaques picked from plates 1 (30cm), 2 (60cm) and 3 (90cm) of the IVTT and viral RNA extracted. The haemagglutinin (HA) gene was Sanger sequenced. HA mutations identified in viral plaques are represented by the colours orange V19I, blue I57F, green T241I and purple L98M-HA2. Lower panel: The proportion of corresponding HA mutations on days 1 to 4 in the nasal wash was determined by next-generation sequencing. In each of the bar graphs, the proportion of virus in nasal wash with sequence encoding the amino acid as in the parental virus (Y7H) is shown in grey. (B) HA mutations are modelled on a HA monomer using Pymol molecular visualization tool (PDB: 4jtv). H1 numbering using the mature HA sequence is used throughout[54]. HA1 is shaded light brown, HA2 is teal and the fusion peptide is black. (C) The acid stability of viruses in air emitted from donor #2 was tested by incubating virus propagated from IVTT plaques at low (pH 5.5) and neutral (pH 7) pH, in triplicate. The remaining infectivity detected at pH 5.5 is expressed relative to infectivity detected at pH 7. Each grey bar represents an individual virus propagated from a picked plaque with its HA mutation on the x-axis. Stability of parental virus Y7H is shown on the left of the panel. Error bars represent standard deviation of three independent experiments. One-way ANOVA with Tukey post-test was used to compare each plaque with the Y7H parent virus. *p<0.05, **p<0.01 ***p<0.001 ****p<0.0001. (PDF) [file ppat.1008362.s005.pdf]

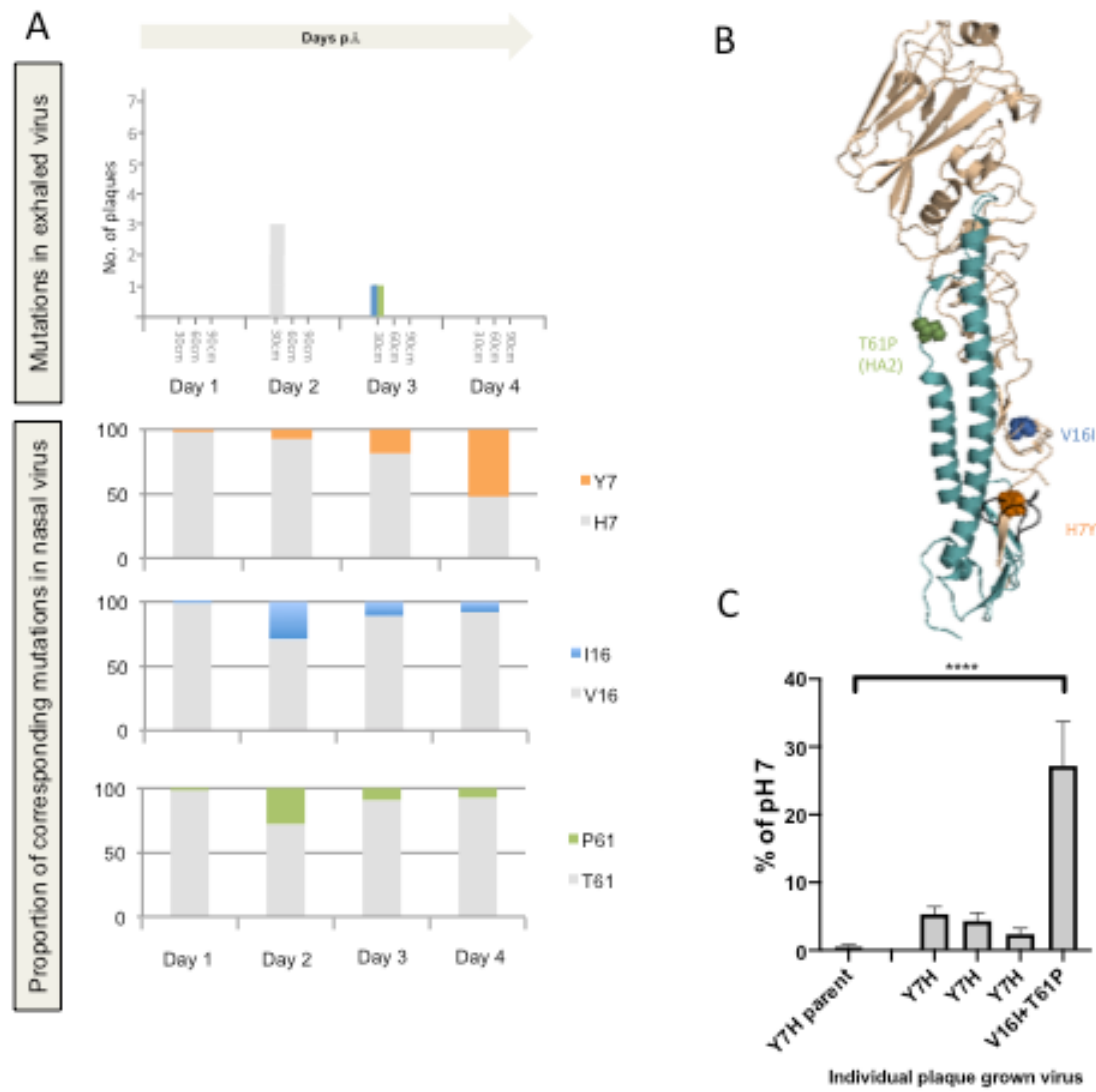

Fig S6

Supplement: S6 Fig — (A) Upper panel: Virus emitted by Y7H-infected ferret donor #4 on days 1 to 4 was collected as picked plaques from plates 1 (30cm), 2 (60cm) and 3 (90cm) of the IVTT and viral RNA extracted. The haemagglutinin (HA) gene was Sanger sequenced. HA mutations identified in viral plaques are represented by the colours orange H7Y, blue V16I, green T61P in HA2. Lower panel: The proportion of corresponding HA mutations at positions 7 and 16 in HA1 and 61 in HA2 detected on days 1 to 4 in the nasal wash was determined by next-generation sequencing. No additional mutations were detected in the nasal wash at >5% frequency. In each of the bar graphs, the proportion of virus in nasal wash with sequence encoding the amino acid as in the parental virus (Y7H) is shown in grey. (B) HA mutations are modelled on a HA monomer using Pymol molecular visualization tool (PDB: 4jtv). H1 numbering using the mature HA sequence is used throughout[54]. HA1 is shaded light brown, HA2 is teal and the fusion peptide is black. (C) The acid stability of viruses in air emitted from donor #4 was tested by incubating virus propagated from IVTT plaques at low (pH 5.5) and neutral (pH 7) pH, in triplicate. The remaining infectivity detected at pH 5.5 is expressed relative to infectivity detected at pH 7. Each grey bar represents an individual virus propagated from a picked plaque with its HA mutation on the x-axis. Stability of parental virus Y7H is shown on the left of the panel. Error bars represent standard deviation of three independent experiments. One-way ANOVA with Tukey post-test was used to compare each plaque with the Y7H parent virus. *p<0.05, **p<0.01 ***p<0.001 ****p<0.0001. (PDF) [file ppat.1008362.s006.pdf]
