# Supplementary material for: Characterising viable virus from air exhaled by H1N1 influenza-infected ferrets reveals the importance of haemagglutinin stability for airborne infectivity
Source: PLoS Pathog. 2020 Feb 25;16(2):e1008362. doi: 10.1371/journal.ppat.1008362 (PMC7059951; doi:10.1371/journal.ppat.1008362)
Supplement: S3 Fig — The Y7H virus inoculum was interrogated for the presence of any low frequency mutations. Data on the amino acid present in the positions at which mutations were detected in IVTT plaques exhaled by Y7H-infected ferrets is shown. (PDF) [file ppat.1008362.s003.pdf]

Amino acid	Position	HA Chain	Frequency (%)
H	7	HA1	100
V	16	HA1	100
V	19	HA1	99.8
N	31	HA1	99.9
R	45	HA1	99.9
I	57	HA1	100
T	241	HA1	99.8
S	289	HA1	99.8
E	47	HA2	99.7
V	55	HA2	100
T	61	HA2	99.8
L	98	HA2	99.8
P	182	HA1	98.5

Fig S3
